# Efficacy and Safety of Anti-PD-1 Plus Anlotinib in Patients With Advanced Non–Small-Cell Lung Cancer After Previous Systemic Treatment Failure—A Retrospective Study

**DOI:** 10.3389/fonc.2021.628124

**Published:** 2021-03-15

**Authors:** Peiliang Wang, Xiaozhuang Fang, Tianwen Yin, Hairong Tian, Jinming Yu, Feifei Teng

**Affiliations:** ^1^Department of Radiation Oncology, Shandong Cancer Hospital and Institute, Cheeloo College of Medicine, Shandong University, Jinan, China; ^2^Department of Radiation Oncology, Shandong Cancer Hospital and Institute, Shandong First Medical University and Shandong Academy of Medical Sciences, Jinan, China; ^3^Department of Hepatobiliary Surgery, Shandong Cancer Hospital and Institute, Cheeloo College of Medicine, Shandong University, Jinan, China

**Keywords:** anlotinib, anti-PD-1, non–small cell lung cancer, combination therapy, immune checkpoint inhibitors

## Abstract

**Background:**

Pre-clinical and clinical evidences support that simultaneous blockade of programmed death-1 (PD-1) and vascular endothelial growth factor receptor (VEGFR) can enhance antigen-specific T-cell migration, and show tolerable toxicity with favorable antitumor activity in patients. In this study, we aimed to assess the safety and efficacy of anlotinib, a novel multitarget tyrosine kinase inhibitor for VEGFR, platelet-derived growth receptor (PDGFR), and the stem cell-factor receptor (c-Kit), combined with anti-PD-1 treatment in patients with advanced NSCLC.

**Methods:**

Sixty-seven patients with previously treated advanced NSCLC receiving anti-PD-1 agents concomitant with anlotinib were retrospectively enrolled in an IRB approved study. Anti-PD-1 agents including pembrolizumab, nivolumab, camrelizumab, toripalimab, sintilimab, and tislelizumab were administered every two or three weeks until disease progression or unacceptable toxicity was reached. Anlotinib was administered orally once daily on days 1–14 of a 21-day cycle. The safety and tolerability of the combination treatment were assessed by the incidence of adverse events. The efficacy of the treatment was assessed by the tumor response and survival.

**Results:**

With a median follow-up period of 8.7 months, treatment-related adverse events occurred in 85% (57/67) of patients and grade 3–4 adverse events were observed in 27 patients (40%). No unexpected adverse events or significantly increased toxicities were observed. Complete response was not observed, 19 patients had partial response (28.4%), 39 had stable disease (58.2%) and 9 had progressive disease (13.4%). The overall response (ORR) and disease control rates (DCR) were 28.4% and 86.6%, respectively. The median progression-free survival (PFS) was 6.9 months (95% CI, 5.5-8.3 months) and overall survival (OS) was 14.5 months (95% CI, 10.9-18.1 months). The benefit of anti-PD-1 plus anlotinib was also observed in patients with EGFR mutation positive, liver metastases and brain metastases.

**Conclusion:**

Anti-PD-1 treatment concomitant with anlotinib has tolerable toxicity and favorable antitumor activity in patients with previously treated advanced NSCLC. Our results add to the growing evidence that supports the benefits of combining immunotherapy with antiangiogenic drugs. This combination could be further evaluated with or without chemotherapy, since no additional toxicity was observed in the combination treatment.

## Introduction

Tumors can evade immune-mediated killing through the interaction between PD-L1 mainly expressed by themselves and PD-1, the inhibitory receptor primarily located on tumor infiltrating T cells, which leads to T cell exhaustion. Immune checkpoint inhibitors (ICIs) targeting the PD-L1–PD-1 axis have shown superior survival outcomes compared with cytotoxic chemotherapies in patients with advanced non–small cell lung cancer (NSCLC) (1–3). Several ICIs targeting PD-1 have been approved by the U.S. Food and Drug Administration (FDA) for the clinical treatment of advanced NSCLC, including durvalumab as consolidation treatment in stage III NSCLC patients (4), pembrolizumab(PD-L1≥1) as a single agent or combined with chemotherapy for first-line treatment of patients with metastatic NSCLC (5, 6), nivolumab, pembrolizumab or atezolizumab as second-line treatment in advanced NSCLC (1, 7, 8). Despite anti-PD-1 or anti-PD-L1 clinical trials producing unprecedented positive clinical outcomes, responses are achieved only in about 20% of unselected patients (8, 9), highlighting the need to identify novel combination treatments that broaden the benefit of anti-PD-1/PD-L1 therapies.

Abnormal tumor vasculature might be one of the mechanisms of resistance to immunotherapy. It can exert immunosuppressive effects including the inhibition the maturation of dendritic cells (DCs), the prevention of T cells infiltration into tumors, and the induction of regulating cells (Tregs) and Myeloid-Derived Suppressor Cells (MDSCs) (10, 11). Substantial data has accumulated showing that antiangiogenic therapies targeting the vascular endothelial growth factor (VEGF) or VEGF receptor-2 (VEGFR-2) can modulate the tumor immunosuppressive microenvironment and might help to reverse resistance to immunotherapy (12–14). A translational study, in Colon-26 adenocarcinoma model, shows that simultaneous blockade of PD-1 and VEGFR enhance ICI-induced effects such as reinforcement of antigen presentation and increase of T cells infiltration (15). In a phase Ia/b trial that assessed the preliminary antitumor activity of ramucirumab (anti-VEGFR2 antibody) combined with pembrolizumab in NSCLC patients, 30% of the patients achieved an objective response, with a median PFS of 9.7 months and a median OS of 26.2 months (16). In a phase III study, a benefit was seen in patients with chemotherapy-naive NSCLC when treated with atezolizumab plus bevacizumab with chemotherapy versus bevacizumab with chemotherapy (OS: HR, 0.78 [95% CI 0.62–0.96]; ORR, 64% vs. 48%, respectively) (17), suggesting the clinical benefit of combining anti-angiogenetics with checkpoint blockades.

Anlotinib (AL3818) hydrochloride is a novel small-molecule inhibitor targeting multiple receptor tyrosine kinases involved in tumor angiogenesis, proliferative signaling and tumor microenvironment (18, 19). Anlotinib mainly inhibits VEGF/VEGFR signaling by selectively targeting VEGFR-2,-3 and the fibroblast growth factor receptors (FGFR-1,-2,-3,-4), and also suppresses the activity of the platelet-derived growth factor receptors α/β (PDGFRα/β), c-FMS c-Kit, Aurora-B, and discoidin domain receptor 1 (DDR1) (20). In phase 3 of the ALTER randomized clinical trial, anlotinib has shown antitumor activity as ≥ 3 lines of treatment in patients with advanced NSCLC, with a prolonged median overall survival (OS) versus placebo (9.6 months for anlotinib vs 6.3 months for placebo; P =0.002) (21). Anlotinib also shows encouraging efficacy and a manageable toxicity in a broad range of malignancies, including soft tissue sarcoma (Clinical Trials.gov: NCT01878448), medullary thyroid cancer (Clinical Trials.gov: NCT01874873), and renal cell cancer (Clinical Trials.gov: NCT02072044). According to the results, anlotinib received its first approval as a third-line treatment for advanced NSCLC and its second approval as a second-line treatment for advanced soft-tissue sarcoma in the People’s Republic of China. At present, although preclinical trials have shown that the combined antiangiogenic and anti-PD-1 therapy has a positive application prospect, the safety and efficacy of anlotinib combined with anti-PD-1 are still unknown.

This study is intended to evaluate the antitumor activity and safety of anti-PD-1 plus anlotinib in advanced NSCLC. We also explored the clinical efficacy of the combination treatments in key subgroups of patients, including patients with EGFR mutations and patients with baseline liver metastases.

## Methods

### Patient Selection and Procedures

We retrospectively enrolled patients with histologically confirmed advanced NSCLC who experienced disease progression after ≥1 systemic treatment. An Eastern Cooperative Oncology Group (ECOG) performance status of 0–2 and measurable disease based on Response Evaluation Criteria in Solid Tumors (RECIST) version 1.1 were also required.

Patients received one of the following anti-PD-1 agents until disease progression, clinical deterioration, or unacceptable toxicity: sintilimab (Innovent Biologics, China), toripalimab (Shangha Merck & Co.), camrelizumab (Jiangsu Hengrui Medicine, China), nivolumab (Bristol-Myers Squibb, USA), pembrolizumab (Merck & Co., USA), or tislelizumab (BeiGene, China). Anlotinib (Chia Tai Tianqing Pharmaceutical, China) was administered orally, once daily (8 mg, 10 mg or 12 mg) on days 1–14 of a 21-day cycle.

The authors are accountable for all aspects of the work in ensuring that questions related to the accuracy or integrity of any part of the work are appropriately investigated and resolved. The study was conducted in accordance with the Declaration of Helsinki (as revised in 2013). The study was approved by the Research Ethics Board of Shandong Cancer Hospital, and individual consent for this retrospective analysis was waived.

### Outcomes

Safety and tolerability was evaluated throughout the study using the National Cancer Institute Common Terminology Criteria for Adverse Events, version 4.0. Measurable disease was assessed and documented before initiating treatment and at least one imaging follow-up had been scheduled for each patient. Radiological assessments of target and non-target lesions were performed every six weeks during the treatment phase until confirmation of disease progression was made. Tumor response was evaluated using RECIST 1.1. Objective tumor responses included complete response (CR), partial response (PR), stable disease (SD), and progressive disease (PD). Progression-free survival (PFS) denoted the time between the first anti-PD-1 dosing day and the documented progression or mortality from any cause. Overall survival (OS) denoted the time between the first anti-PD-1 dosing day and mortality or the last follow-up.

### Statistical Analyses

Survival analyses were performed using the Kaplan-Meier method and the comparison of survival times was performed using the log-rank test. Univariate and multivariate analyses were conducted using the Cox proportional hazards model to analyze factors associated with treatment response and survival. Covariates with p values <0.1 on univariate analyses were incorporated in the multivariate model, which was constructed using the enter method. All other statistical analyses were performed using SPSS 24.0 (IBM, Armonk, NY, USA), and a p-value of <0.05 was considered statistically significant.

## Results

### Patients and Treatment

A total of 67 consecutive patients were enrolled between August, 2018 and September, 2020. Baseline demographic and clinical characteristics are listed in **Table 1**. The median age was 60 years (range: 33 to 77 years), and 47 of the patients (70%) were males. More than three quarters of the patients (56 patients, 84%) were diagnosed as having stage IV and more than half of the patients (38 patients, 57%) had >3 metastatic sites. 41 patients (61%) were diagnosed with adenocarcinoma. Among the 39 patients whose dates of EGFR testing were available, nine (23%) patients were positive for EGFR mutation. Unfortunately, the PD-L1 status was only assessed in nine patients, since the biopsy samples were not sufficient in most patients. Presence of liver metastases at baseline was reported in 18 (27%) patients and 16 (24%) patients had brain metastases. Of the 67 patients, 21 (31%) received previous first-line systemic therapy, whereas 46 (69%) received previous second- or further-line systemic therapy. Sintilimab (28 patients), toripalimab (13 patients), and camrelizumab (12 patients) were the three main anti-PD-1 drugs, accounting for 79% of the total population.

**Table 1:** Baseline Characteristics of Study Population.

Characteristic	Patients (N=67)
Age, median (range, year)	60 (33-77)
Sex, n (%)	
Male	47 (70%)
Female	20 (30%)
ECOG performance status, n (%)	
0	19 (28%)
1-2	48(72%)
Smoking status, n (%)	
≥10 pack-years	32 (48%)
<10 pack-years	35 (52%)
Histology, n (%)	
Squamous	26 (39%)
Adenosquamous	41 (61%)
Surgery treatment, n (%)	
Yes	24 (36%)
No	43 (64%)
Stage	
III	11 (16%)
IV	56 (84%)
Anlotinib dose, n (%)	
8mg	7 (10%)
10mg	32 (48%)
12mg	28 (42%)
EGFR mutation, n (%)	
EGFR(+)	9 (13%)
EGFR(-)	30 (45%)
Unknown	28 (42%)
PD-L1 status, n (%)	
Positive(TPS≥1%) Negative(TPS<1%)	4 (6%) 5 (7%)
Not reported	58 (87%)
Liver metastases, n (%)	
Absent	49 (73%)
Present	18 (27%)
Brain metastases, n (%)	
Absent	51 (76%)
Present	16 (24%)
Metastatic sites, n (%)	
≤3	29 (43%)
>3	38 (57%)
Previous systemic therapy, n (%)	
1	21 (31%)
≥2	46 (69%)
Anti-PD-1drugs	
sintilimab	28 (42%)
toripalimab	13 (19%)
camrelizumab	12 (18%)
nivolumab	7 (10%)
tislelizuma	4 (6%)
pembrolizumab	3 (4%)

ECOG, Eastern Cooperative Oncology Group; EGFR, epidermal growth factor receptor; TPS, tumor proportion score.

### Safety

The overall incidence of adverse events was 85% (57 of 56), and most of these observed adverse events were grade 1–2 (**Table 2**). Grade 3–4 treatment-related adverse events occurred in 27 patients (40%). No fatal adverse events were observed. 8 patients (12%) underwent anlotinib dose modification due to adverse events. These grade 3–4 adverse events were hypertension (12 patients, 18%), transaminitis (6 patients, 9%), diarrhea (4 patients, 6%), hypothyroidism (4 patient, 6%), hand-foot syndrome (3 patients, 4%), mouth ulceration (3 patients, 4%), headache/dizziness (1 patients, 1%), rash (1 patients, 1%), neutropenia (1 patients, 1%), and thrombocytopenia (1 patients, 1%). The combination of anti-PD-1 and anlotinib was safe, with no new toxicity signals compared with monotherapy (**Table 2**).

**Table 2:** Treatment-Related Adverse Events with at Least 10% Incidence in Study Population.

	No. (%) of Patients (n = 67)		
	All grades	Grades 1-2	Grades 3-4
Any adverse event	57(85)	49(73)	27 (40)
Hypertension	40(60)	28 (42)	12 (18)
Fatigue	37 (55)	37(55)	0
Transaminitis	36 (54)	30 (45)	6 (9)
Diarrhoea	20 (30)	16 (24)	4 (6)
Headache/Dizziness	18 (27)	17 (25)	1(1)
Rash	14 (21)	13(19)	1 (1)
Neutropenia	14 (21)	13 (19)	1 (1)
Nausea	13 (19)	13 (19)	0
Cough	12 (18)	12 (18)	0
Hand-foot syndrome	11 (16)	8 (12)	3 (4)
Proteinuria	10 (15)	10 (15)	0
Pruritus	9 (13)	9 (13)	0
Dyspnea	9 (13)	9 (13)	0
Hypothyroidism	9 (13)	5 (7)	4 (6)
Thrombocytopenia	8 (12)	7(10)	1 (1)
Mouth ulceration	7 (10)	4 (6)	3 (4)

### Efficacy

**Figure 1:**
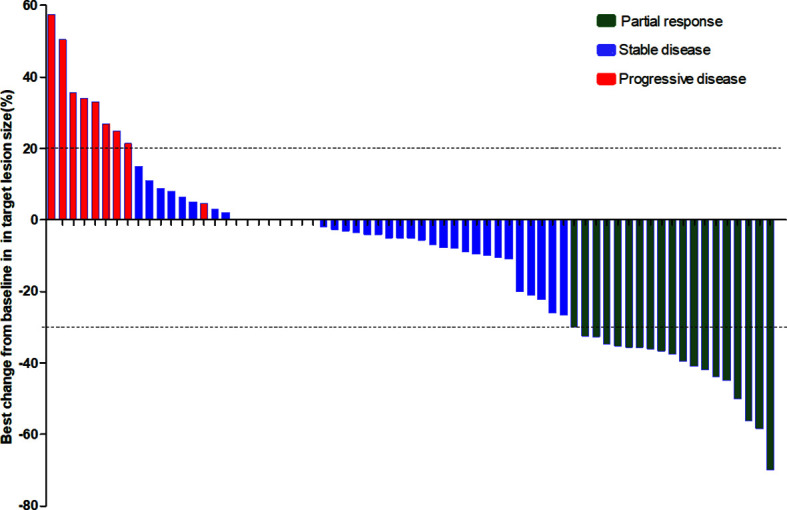
Waterfall plot illustrating maximum change in target lesion size (N = 56).

**Figure 2:**
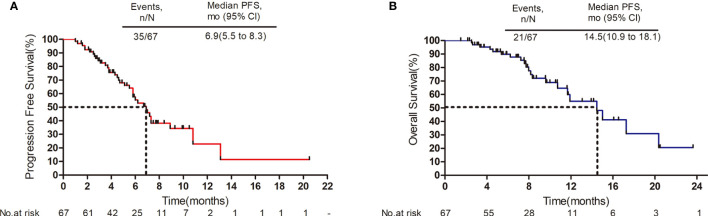
Survival outcomes. **(A)** Progression-free survival. **(B)** Overall survival. Mo, months; CI, confidence interval.

The median follow-up period was 8.7 months (range: 1.5 to 23.6 months). As shown in the waterfall plot (**Figure 1**), 19 patients obtained PR, 39 patients exhibited SD, 9 patients developed PD, and none of the patients achieved CR, yielding an overall response rate (ORR) of 28.4% and disease control rate (DCR) of 86.6%. The maximum percent change in target lesion size from the baseline was -70% (**Figure 1**). The median PFS was 6.9 months (95% CI 5.5–8.3 months), and median OS was 14.5 months (95% CI, 10.9–18.1 months) (**Figures 2A, B**). In the full analysis set, death occurred in 21 (31%) patients by the cutoff date; 46 (69%) patients were alive and 26 (39%) patients were being treated at the time of analysis.

**Figure 3:**
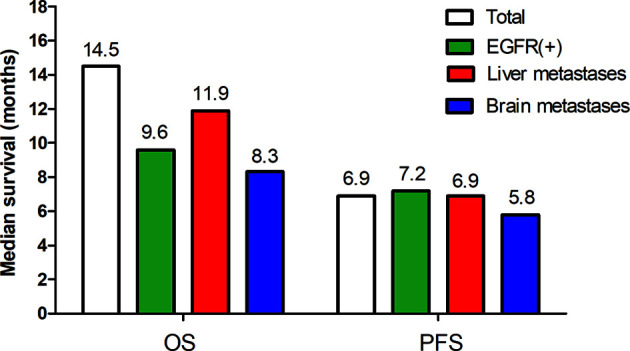
Survival outcomes for key subgroups (EGFR positive, liver metastases, brain metastases).

Based on the key subgroup analyses of patients with EGFR mutations, baseline liver metastases or brain metastases, the median PFS was 7.2 months, 6.9 months, and 5.8 months respectively; the median OS was 9.6 months, 11.9 months, and 8.3 months respectively (**Figure 3**).

We also performed exploratory analyses to determine whether any clinical or pathologic features were associated with PFS and OS. In univariate cox analysis, histology and metastatic sites were associated with PFS (p=0.049, p=0.018 respectively) (**Table 3**); Metastatic sites (p=0.006) and metastases brain (p=0.024) were significantly associated with OS (**Table 4**). The number of previous systemic therapies, anlotinib dose, ECOG performance status, and TN stage and liver metastases status were not found to be associated with any predictive effects. In multivariate analysis, only the number of metastatic sites was found to independently predict PFS and OS (**Tables 3**, **4**). Patients with < 3 metastatic sites showed better survival to the combination treatment (PFS: HR, 2.267; 95% CI, 1.084–4.742; p=0.030; OS: HR, 3.474; 95% CI, 1.193–10.113; p=0.022).

**Table 3:** Univariate and Multivariate Cox Regression Analysis of Factors Associated with PFS.

Characteristics	Univariate analysis			Multivariate analysis		
	HR	95% CI	p value	HR	95% CI	p value
Age (≤60 vs >60)	1.253	0.643-2.443	0.508			NI
Gender (Male vs Female)	1.224	0.571-2.627	0.603			NI
Smoking (<10 pack-years vs ≥10 pack-years)	0.772	0.397-1.499	0.444			NI
ECOG performance status (1-2 vs 0)	1.544	0.780-3.097	0.210			NI
Histology (Squamous vs Adenosquamous)	0.745	0.373-1.489	0.405			NI
Surgery treatment (Yes vs No)	0.483	0.234-0.997	**0.049**	0.526	0.254-1.090	0.084
T Stage (T1-2 vs T3-4)	1.244	0.40-2.417	0.520			NI
N stage (N0-1 vs N2-3)	0.451	0.157-1.289	0.137			NI
Previous systemic therapy (1 vs ≥2)	0.969	0.469-2.005	0.933			NI
Anlotinib dose (8mg/10mg vs 12mg)	1.146	0.859-1.530	0.354			NI
Metastatic sites (>3 vs ≤3)	2.431	1.166-5.066	**0.018**	2.267	1.084-4.742	**0.030**
Liver metastases(Absent vs Present)	0.821	0.381-1.769	0.615			NI
Brain metastases(Absent vs Present)	0.702	0.341-1.565	0.387			NI

ECOG, Eastern Cooperative Oncology Group; EGFR, epidermal growth factor receptor; HR, hazard ratio; CI, confidence interval; NI, not included in multivariate model; Boldness indicates p-value less than 0.05.

**Table 4:** Univariate and Multivariate Cox Regression Analysis of Factors Associated with OS.

Characteristics	Univariate analysis			Multivariate analysis		
	HR	95% CI	p value	HR	95% CI	p value
Age (≤60 vs >60)	0.638	0.260-1.562	0.325			NI
Gender (Male vs Female)	0.740	0.298-1.838	0.516			NI
Smoking (<10 pack-years vs ≥10 pack-years)	1.056	0.434-2.570	0.905			NI
ECOG performance status (1-2 vs 0)	1.691	0.709-4.033	0.236			NI
Histology (Squamous vs Adenosquamous)	1.448	0.599-3.496	0.411			NI
Surgery treatment (Yes vs No)	0.667	0.267-1.666	0.386			NI
T Stage (T1-2 vs T3-4)	0.856	0.343-2.139	0.740			NI
N stage (N0-1 vs N2-3)	0.904	0.325-2.513	0.847			NI
Previous systemic therapy (1 vs ≥2)	1.520	0.548-4.212	0.421			NI
Anlotinib dose (8mg/ 10mg vs 12mg)	0.945	0.683-1.308	0.732			NI
Metastatic sites (>3 vs ≤3)	4.178	1.510-11.558	**0.006**	3.474	1.193-10.113	**0.022**
Liver metastases(Absent vs Present)	0.924	0.331-2.577	0.880			NI
Brain metastases(Absent vs Present)	0.342	0.125-0.871	**0.024**	0.551	0.201-1.513	0.247

ECOG, Eastern Cooperative Oncology Group; EGFR, epidermal growth factor receptor; HR, hazard ratio; CI, confidence interval; NI, not included in multivariate model; Boldness indicates p-value less than 0.05.

## Discussion

In the present study, anti-PD-1 treatment concomitant with anlotinib has tolerable toxicity and favorable antitumor activity in patients with previously treated advanced NSCLC. As a potential effective treatment regimen, some clinical trials are underway to assess the efficacy of the combination of checkpoint inhibitors with anti-angiogenetics. Our results provided more evidence for the following clinical trials.

Immune checkpoint inhibitors as second or third-line monotherapy has shown limited therapeutic benefit in patients with NSCLC. In the CheckMate 057 (22) and KEYNOTE-001 (8) studies, the ORRs of anti-PD-1 monotherapy were 19% (median PFS of 2.3 months) and 19.4% (median PFS of 3.7 months), respectively. Our results demonstrated the efficacy of anti-PD-1 plus anlotinib, as shown by the ORR of 28.4% and DCR of 86.6%, with a median PFS of 6.9 months (95% CI, 5.5-8.3 months), which was superior to that of anti-PD-1 monotherapy in the second-line setting. The most common toxic effects for the anti-PD-1 plus anlotinib combination therapy were of grade one or two severity, with few patients discontinuing treatment due to adverse events. Although the proportion of patients having grade 3–4 adverse events was higher than that previously reported for anti-PD-1 monotherapy (40% vs 7-10%) (7, 8, 22), most of these events did not affect treatment or could be resolved.

The use of immune checkpoint inhibitors (anti-PD-1 or anti-PD-L1) as monotherapy has shown poor outcome in patients with EGFR mutations (3, 23). Data from IMpower150 showed that the combination of atezolizumab, bevacizumab, carboplatin, and paclitaxel provided OS and PFS benefits to patients with sensitizing EGFR mutations compared to patients who received the standard-of-care bevacizumab, carboplatin, and paclitaxel regimen (24). In our study, the EGFR-positive group had a mPFS of 7.2 months and a mOS of 9.6 months. Immune checkpoint inhibitor monotherapy has also shown minimal therapeutic benefit in patients with liver metastases—a common metastatic site for NSCLC and a negative prognostic indicator (25–27). According to our results, the patients with liver metastasis had a mPFS of 6.9 months and a mOS of 11.9 months. In addition, there is a paucity of data on anti-PD-1 plus antiangiogenesis therapy among patients with brain metastases. Our results indicated that median PFS was 5.8 months, and median OS was 8.3 months in patients with brain metastases. Whether the clinical benefit can extend across these subgroups with EGFR genetic alterations, baseline liver metastases or brain metastases should be further studied in future randomized trials.

The limitation of immunotherapy in solid tumors is the activation of multiple immunosuppressive components in the tumor microenvironment (28). A low level expression of the PD-L1 in tumors alone cannot explain the lack of responsiveness in the majority of patients, nor can a low number of tumor mutational burden (TMB). The VEGF-VEGFR signaling can contribute to local and systemic immunosuppression through a variety of mechanisms. The excessive activation of VEGF-VEGFR pathways can directly inhibit the trafficking of immune cells to the tumor by inhibiting upregulation of the expression of intercellular adhesion molecule-1(ICAM-1) and vascular cell adhesion molecule-1(VCAM-1) (11, 29). In addition, VEGF also reprogrammed the immunosuppressive microenvironment through various mechanisms, such as boosting immunosuppressive cytokines (IL-10, TGF β), enhancing expression of inhibitory checkpoints (such as PD1, CTLA4, and LAG-3) in CD8^+^ T cells, and increasing the presence of MDSCs and Treg (30, 31). Thus, antiangiogenics that normalize the tumor microenvironment could potentially improve immunotherapy effectiveness. This was confirmed in a pre-clinical study, which suggested that the application of anti-VEGF-A antibody (sunitinib) in CT26 tumor-bearing mice increases the infiltration of cytotoxic tumor-infiltrating lymphocytes (TIL) and decreases PD-1 expression in CD8^+^T cells (32). Moreover, another research provided evidence that anti-PD-L1 therapy, in reverse, can make tumors sensitive to antiangiogenic therapy and improve its efficacy (33).

Our study has some limitations. The retrospective nature and relatively small sample size were two major limitations, which mean selection bias could not be ruled out. Given that this study was a single-arm study, we could not formally establish the role of the combination therapy over anti-PD-1 monotherapy. Additionally, we enrolled a heterogeneous patient population treated using a variety of anti-PD-1 drugs and did not have a study design with sufficient power for subgroup analyses with respect to PD-L1 status. Date on survival in specific subgroup (EGFR, liver metastasis, brain metastasis) was only descriptive due to the small sample size. However, our study’s findings may still be deemed as meaningful due to the limited number of similar prospective clinical studies in the literature.

Although the combination of antiangiogenic therapy and immunotherapy has been proved to be a very promising treatment in many solid tumors, some issues must be addressed prior to clinical practice application. It is important to identify the optimal dosing and timing to make the combination therapy more effective. Moreover, the exploration of predictive biomarkers is helpful in screening which cancer types and stages would benefit more from this treatment.

## Conclusion

Our findings show that the combination of anlotinib and anti-PD-1 drugs has promising efficacy and manageable toxic effects as a second- or further-line treatment for patients with previously treated advanced NSCLC. The results further demonstrate the clinical applicability of dual inhibition of the VEGF-VEGFR2 and PD-1-/PD-L1 pathways. Given these findings, prospective investigation is warranted to explored with or without chemotherapy, particularly for patients with tumors for which immune checkpoint inhibitor monotherapy was not superior to chemotherapy.

## Data Availability Statement

The original contributions presented in the study are included in the article/supplementary material. Further inquiries can be directed to the corresponding author.

## Ethics Statement

The study was approved by the Research Ethics Board of Shandong Cancer Hospital, and individual consent for this retrospective analysis was waived.

## Author Contributions

Conception and design: PW, FT, and JY. Provision of study materials or patients: PW. Collection and assembly of data: PW, XF, TY, and HT. Data analysis and interpretation: PW and FT. Manuscript writing and final approval of manuscript: all authors. All authors contributed to the article and approved the submitted version.

## Funding

This work was supported by grants from the National Natural Science Foundation of China (NSFC 81803066) and the Innovation Project of Shandong Academy of Medical Sciences (2019-04 to JY), and the Academic Promotion Program of Shandong First Medical University (2019ZL002 to JY).

## Conflict of Interest

The authors declare that the research was conducted in the absence of any commercial or financial relationships that could be construed as a potential conflict of interest.
